# Comparison of peri-implant bone loss between conventional drilling with irrigation versus low-speed drilling without irrigation

**DOI:** 10.4317/medoral.21694

**Published:** 2017-10-21

**Authors:** Hilario Pellicer-Chover, David Peñarrocha-Oltra, Amparo Aloy-Prosper, José-Carlos Sanchis-Gonzalez, María Peñarrocha-Diago, Miguel Peñarrocha-Diago

**Affiliations:** 1 DDS. Collaborating Professor of the Master in Oral Surgery and Implant Dentistry. Oral Surgery Unit, Department of Stomatology, Faculty of Medicine and Dentistry, University of Valencia, Spain; 2 PhD, DDS. Assistant Professor of Oral Surgery Unit, Department of Stomatology, Faculty of Medicine and Dentistry, University of Valencia, Spain; 3 PhD, DDS. Associate Professor of Oral Surgery, Stomatology Department, Faculty of Medicine and Dentistry, University of Valencia, Spain; 4 DDS. Master in Oral Surgery and Implant Dentistry, Oral Surgery Unit, Department of Stomatology, Faculty of Medicine and Dentistry, University of Valencia, Spain; 5 MD, PhD, DDS. Full Professor of Oral surgery Unit, Department of Stomatology, Faculty of Medicine and Dentistry, University of Valencia, Spain; 6 MD, PhD. Chairman of Oral Surgery and Director of the Master in Oral Surgery and Implant Dentistry, Oral surgery Unit, Department of Stomatology, Faculty of Medicine and Dentistry, University of Valencia, Spain

## Abstract

**Background:**

To compare the technique of high speed drilling with irrigation and low speed drilling without irrigation in order to evaluate the success rate and peri-implant bone loss at 12 months of follow-up.

**Material and Methods:**

A randomized, controlled, parallel-group clinical trial was carried out in patients requiring dental implants to rehabilitate their unitary edentulism. Patients were recruited from the Oral Surgery Unit of the University of Valencia (Spain) between September 2014 and August 2015. Patients who met the inclusion criteria were randomized to two groups: group A (high-speed drilling with irrigation) and group B (low-speed drilling without irrigation). The success rate and peri-implant bone loss were recorded at 12 months of follow-up.

**Results:**

Twenty-five patients (9 men and 16 women) with 30 implants were enrolled in the study: 15 implants in group A and 15 implants in group B. The mean bone loss of the implants in group A and group B was 0.83 ± 0.73 mm and 0.62 ± 0.70 mm, respectively (*p* > 0.05). In the maxilla, the bone loss was 1.04 ± 0.63 mm in group A and 0.71 ± 0.36 mm in group B (*p* > 0.05), while bone loss in the mandible was 0.59 ± 0.80 mm in group A and 0.69 ± 0.77 mm in group B (*p* > 0.05). The implant success rate at 12 months was 93.3% in group A and 100% in group B.

**Conclusions:**

Within the limitations of the study, the low-speed drilling technique presented peri-implant bone loss outcomes similar to those of the conventional drilling technique at 12 months of follow-up.

** Key words:**Low-speed without irrigation, drilling technique.

## Introduction

The conventional drilling technique at 1500 rpm described by Bränemark is currently the gold standard in most of implant systems (1). Recently, a new concept of low-speed drilling (50 rpm) without irrigation has been suggested as an alternative to the conventional procedure.

Many studies (2-6) have shown temperature alterations in bone during osteotomy, and significant temperatures increases can result in heat-induced bone injury (7). Calvo-Guirado *et al.* (8) evaluated a new hybrid drilling protocol involving the analysis of thermal changes in vitro and their effects on crestal bone loss and bone-to-implant contact *in vivo*. They found that the new hybrid protocol for preparation of the implant bed without irrigation increases the temperature in a way similar to the conventional incremental protocol. Crestal bone loss and bone-to-implant contact with the new drilling protocol were comparable to those of the conventional drilling protocol, and did not affect the osseointegration process *in vivo*.

Different bone models based on cadaveric bone blocks from cow or pig have been used to record temperatures during drilling (9-12). Three studies used synthetic blocks (13-15) and one used a resin model (16). To our knowledge, no clinical study has compared the peri-implant bone reaction in conventional drilling with irrigation versus low-speed drilling without irrigation. For these reasons, the effects of the drilling technique upon bone healing around dental implants remain unclear. The objective of this study was to compare the techniques of high speed drilling with irrigation versus low speed drilling without irrigation, in order to evaluate the success rate and peri-implant bone loss at 12 months of follow-up.

## Material and Methods

-Patient screening and recruitment

A randomized, controlled, parallel-group clinical trial was carried out involving patients requiring dental implants to rehabilitate their unitary edentulism. Patients were recruited from the Oral Surgery Unit of the University of Valencia (Spain) between September 2014 and August 2015.  Table 1 shows the inclusion and exclusion criteria. The investigation was performed following the principles outlined in the Declaration of Helsinki, and the patients were required to sign a consent form after being fully informed about the study. The study protocol was approved by the Institutional Review Board of the University of Valencia (H1365580155510).

**Table 1:**
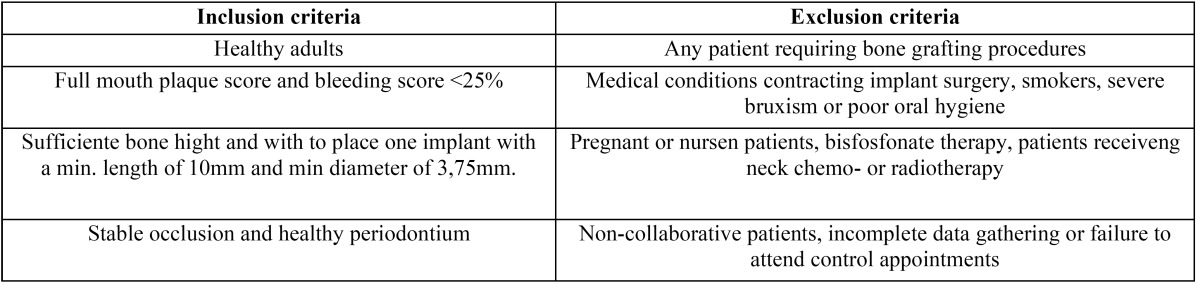
Patient inclusion and exclusion criteria.

-Preoperative procedure

Upper and lower alginate impressions were taken from each patient for planning and fabricating measurement stents and surgical guides. Cone-beam computed tomography (CBCT) (Picasso Master 3D®, Ewoo Technology, South Korea) was performed in all patients after placing a radiological stent marked with radiopaque material at the center of the missing tooth, in order to determine the exact implant site. Imaging was performed with a resolution of 0.1 voxels vision vision, a field-of-view (FOV) of 20 cm x 19 cm, and a slice interval of 1 mm.

Bone density was calculated using the cross-section determined by the radiopaque marker, designating a study area of the same dimensions as the implant according to the indications of Turkyilmaz *et al.* (17), and obtaining measurements of the bone density in that area with Ez-implant® software (Vatech, Yongin-Si, South Korea), expressed in pixels (PV) as described by Song et al. (18). All patients received professional prophylaxis two weeks before surgery, and were given instructions for improving and maintaining oral hygiene at home. The patients received a removable, provisional prosthesis during the healing phase.

After six months of tooth socket healing, each patient was randomized to one of two treatment regimens: group A (conventional high-speed drilling technique with irrigation) or group B (low-speed drilling technique without irrigation). Random assignment was performed by a professional statistician using pre-defined randomization tables. A balanced random permuted-block approach was used to prepare the randomization tables, in order to avoid unequal balance between the two treatment groups. Participants were informed about treatments, but blinded to assignment.

-Surgical procedure

All surgeries were performed under local anesthesia (4% articaine with 1:100,000 adrenalin [Inibsa®, Lliça de Vall, Barcelona, Spain]). Ticare® Inhex® implants (Mozo-Grau, S.L. Valladolid, Spain) were used in the present study, presenting a neck design with microthreads, a rough surface up to the implant platform, a conical connection and platform switching. All patients were treated following a two-step procedure. Calibrated drills with stoppers were used to prepare the sites to the implant length at 800 rpm with saline irrigation (group A) or at 50 rpm without irrigation (group B). The drill sequence used was: initial lance drill, followed by 2.0 mm, 3.0 mm, 3.3 mm and 3.8 mm conical drills. After implant placement and suturing, each patient received 500 mg of amoxicillin (19) (Clamoxyl®, GlaxoSmithKline, Madrid, Spain) three times daily for 7 days, 600 mg of ibuprofen (Bexistar®, Laboratorio Bacino, Barcelona, Spain) to be taken as needed, and a 0.12% chlorhexidine mouthwash (GUM®, John O. Butler/Sunstar, Chicago, IL, USA) for use twice daily during two weeks. Gentle brushing was also recommended. Sutures were removed 8-10 days after surgery. Prosthetic loading was carried out 8 weeks following implant placement.

All the restorations were metal-ceramic and screwed directly to the implant, and were designed and fabricated by the same dental technician. All the structures were designed by the same dental technician and CAD-CAM drilled out of chromium-cobalt at the facilities of the implant manufacturer (Bio-CAM, Mozo-Grau, S.L., Valladolid, Spain). The same dental technician then provided the feldspatic ceramic veneering [IPS d.SIGN, Ivoclar Vivadent, Schaan, Liechtenstein]. All screws were tightened with a torque of 30 Ncm according to the specifications of the manufacturer. The access hole of the screw-retained crowns was closed with a teflon pellet and a hybrid resin composite [Tetric-Ceram, Ivoclar Vivadent, Schaan, Liechtenstein].

-Measurements

A previously established standard protocol was used to compile the following data for all patients: sex, age (at implant placement), implant length, implant diameter, and tooth brushing frequency.

The definition of implant success was based on the clinical and radiographic criteria described by Buser *et al.* (20): 1) absence of clinically detectable implant mobility; 2) absence of pain or any subjective sensation; 3) absence of recurrent peri-implant infection; and 4) absence of persistent radiotransparency around the implant after 12 months of loading.

**Figure 1:**
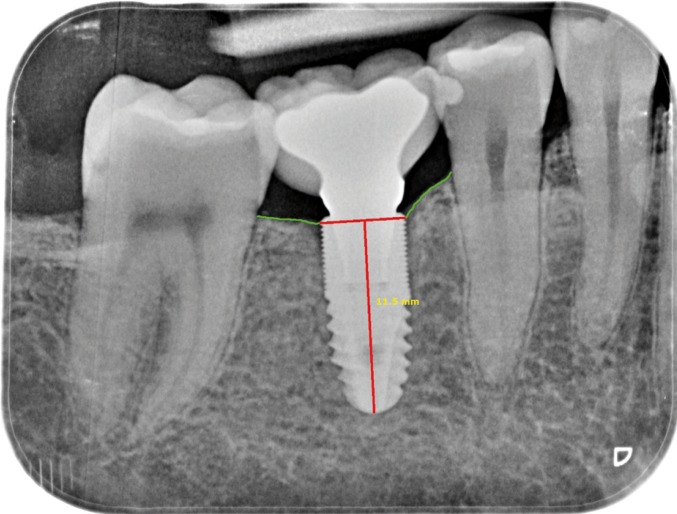
Measurement of peri-implant bone loss with periapical X-ray view after 12 months of follow-up.

Radiological evaluation was carried out at the time of implant placement (T1) and 12 months after loading (T2) using an XMIND intraoral system (GroupeSatelec-Pierre Rolland, Bordeaux, France) and an RVG intraoral digital receptor (Dürr Dental, Bietigheim-Bissingen, Germany). To reproduce the patient alignments, a rigid cross-arch bar was used with bite-registration material, and a Rinn XCP (Dentsply, Des Plaines, IL, USA) rod and ring were firmly attached to the bar and placed in contact with the X-ray cone. The receptor was held by a slot in the bar. Two trained clinicians worked together to interpret the radiographs. Implant marginal bone level was measured to the closest 0.1 mm using DBSWIN software (Dürr Dental, Bietigheim-Bissingen, Germany). For measurement purposes, two visible and easily localized reference points were selected at the implant platform. A straight line was traced joining the two reference points and was taken to represent zero height. For the determination of bone level, a perpendicular line was traced mesial and distal to the implant from zero height to contact with the bone (Fig. 1). The difference between the values recorded at T1 and T2 was used to calculate bone loss mesial and distal to the implant. The average between mesial and distal was selected as the bone loss for the fixation in question.

-Statistical analysis

A generalized estimating equation (GEE) linear model was used to evaluate differences in bone loss and in implant exposed surface, with measurement time as intra-implant factor and the group or type of edentulism as inter-implant factor. The main effects and interactions were studied using the Wald Chi2 statistic. Multiple comparisons were made with Bonferroni correction. For a generalized linear model with a significance level of 5% (*p*=0.05), and considering a mean detected effect size (f=0.25), the statistical power is 0.81 for null contrasting of the interaction.

## Results

**Figure 2:**
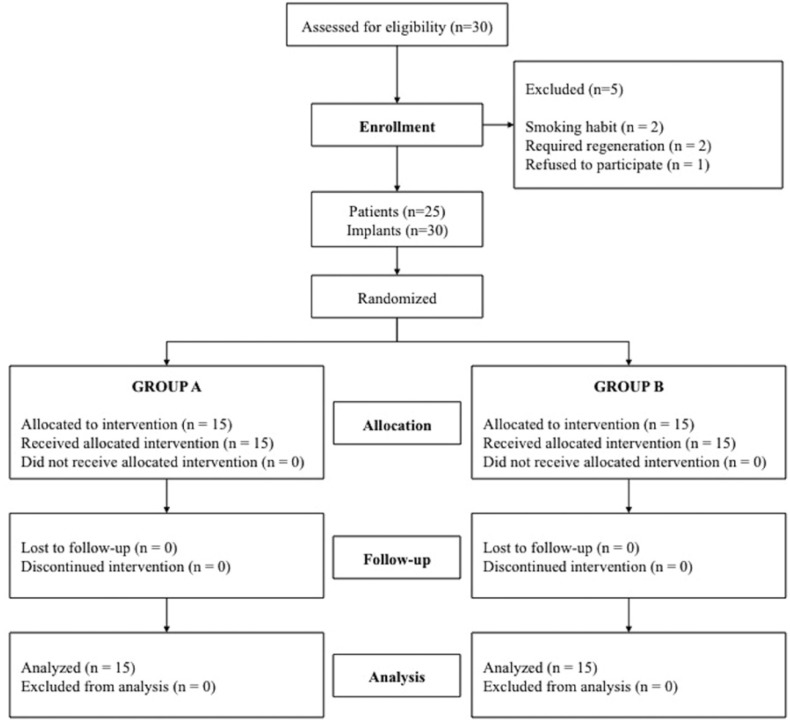
Flow diagram of the study design.

Thirty consecutive patients with the required type of edentulism were screened for the study. Five patients failed to meet the required criteria and were not included (two smoked >10 cigarettes/day; two required regeneration procedures; and one patient declined to participate). A flow diagram of the activities during the phases of the study is shown in Figure 2. Twenty-five patients (9 men and 16 women) between 27 and 80 years of age (mean 50.2 years) were considered eligible and were consecutively enrolled. Patient demographic and implant characteristics are listed in  Table 2. No dropouts occurred during the entire follow-up period. Each patient could have one or more implants placed, according to the inclusion criteria. A total of 30 implants were placed: 15 allocated to each group. All patients completed follow-up and were analyzed. One implant failed during the osseointegration phase in group A; the success rate was therefore 93.3% in group A and 100% in group B.

**Table 2:**
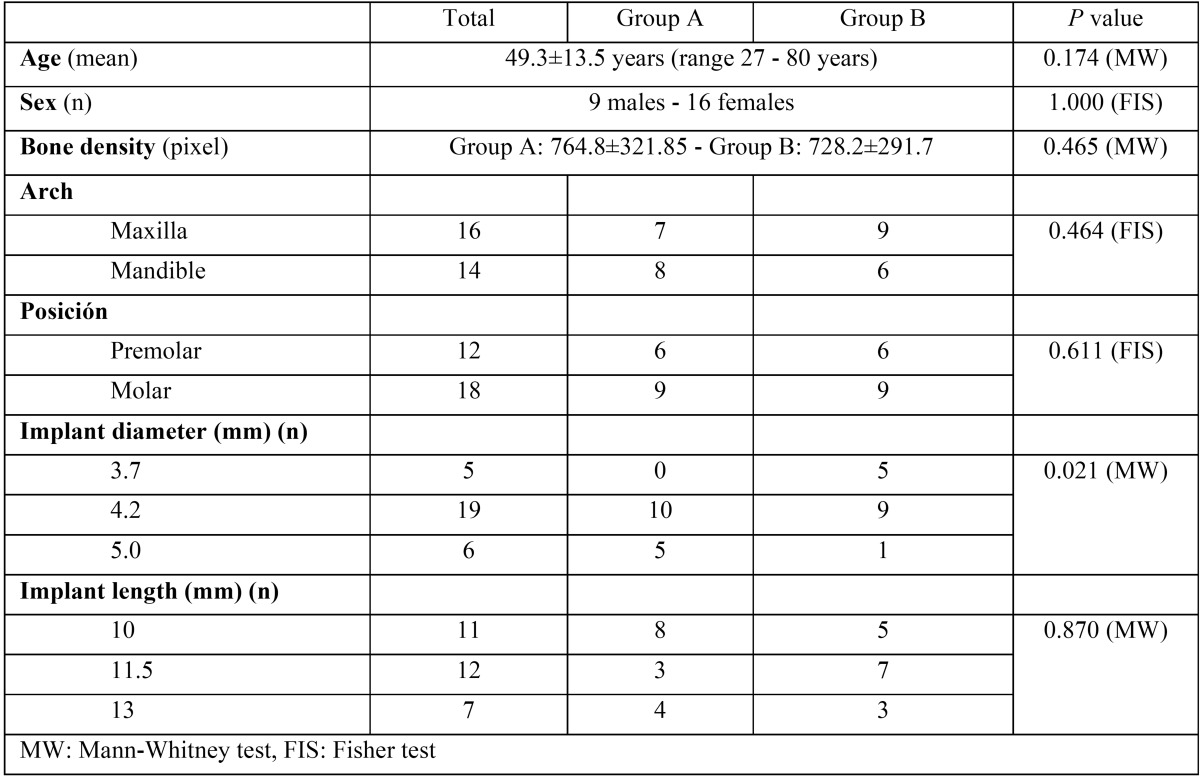
Patient demographic information and implant dimensions.

**Figure 3:**
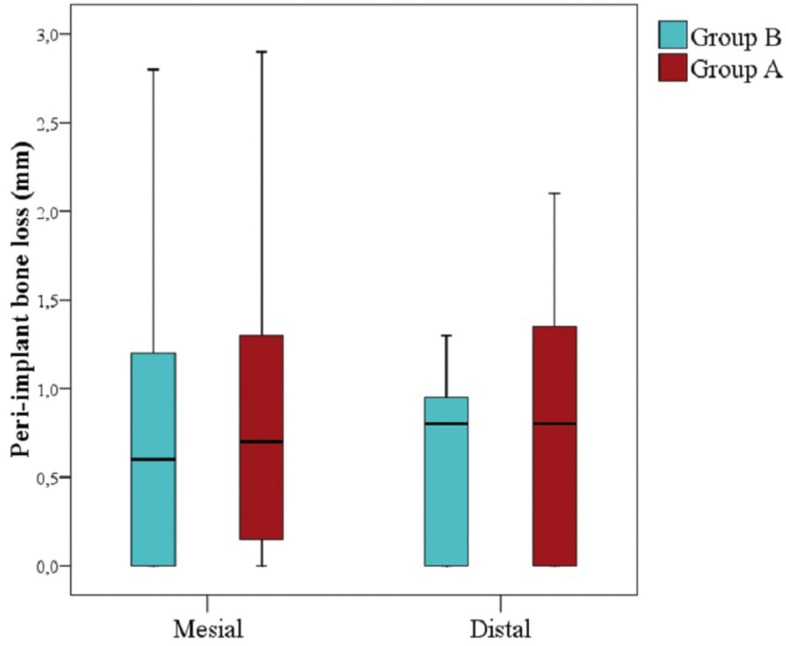
Box plot of mesial and distal peri-implant bone loss in both groups.

 Table 3 shows peri-implant bone level measurements of group A and group B implants at the different peri-implant points. At 12 months of follow-up, the mean bone loss of implants in group A and group B was 0.83±0.73 mm and 0.70±0.62 mm, respectively, with no significant difference between groups (*p*=0.458) (Fig. 3). With regard to implants placed in the maxilla, group A and group B showed a peri-implant bone loss of 0.59 ± 0.80 mm and 0.69 ± 0,77 mm respectively, versus 1.04 ± 0.63 mm and 0.71 ± 0.36 mm in the mandible.

**Table 3:**
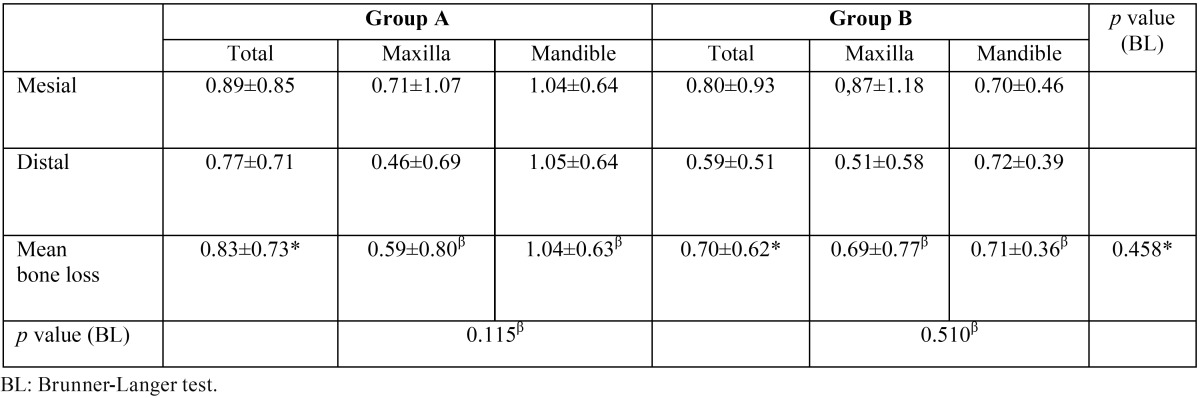
Mesial, distal and mean measurements of peri-implant bone loss at 12 months of follow-up in groups A and B.

## Discussion

The aim of this study was to compare conventional drilling (at high speed with irrigation) versus low speed drilling without irrigation. One implant belonging to the irrigation group failed in our study - no relationship being observed between the technique applied and implant survival.

One of the main concerns of implant placement in drilling without irrigation is the temperature rise caused by drill friction in the bone. Using fixed thermal chambers, Eriksson and Albrektsson (7-21) investigated the histological effects of heat upon bone. When a temperature rise occurred, arterial and venous hyperemia was observed during acute effects, and blood flow stopped in different parts of the capillary network. There were no connective tissue reactions. A chronic effect was characterized by recirculation of the capillaries after four days, and was associated to slight elongation of the vessels. Adipose cells began to reabsorb two days after the thermal increase and continued to do so for 14 days. They changed in shape and color, and new fat cells were produced. At the third week about 30% of the bone had been resorbed. The authors concluded that in order to enable successful osseointegration of endosseous implants, low temperatures are required during preparation of the recipient site.

Different factors affect the heat generated during drilling at the implant site, including the operator (pressure, status, movement, speed and duration of drilling), manufacturer (design and sharpness of the drill, irrigation system and implant system), site (cortical thickness, condition of the site and depth drilled) and patient (age and bone density) (22). In an animal study, Trisi *et al.* (23) analyzed histomorphometric parameters in implants that had been placed with different irrigation systems (without irrigation, with internal irrigation, with external irrigation, and a combination of both). The results of the study suggest that due to insufficient irrigation, hard bone caused massive resorption of the cortical bone and implant failure. In all study groups, the drilling speed was 1000 rpm, justifying thermal injury in the group of non-irrigated implants. Sarendranath *et al.* (24), in a study in dogs, compared conventional drilling with a simplified drilling technique at 400 rpm with irrigation. The results suggest that the simplified procedure yields biological outcomes comparable to those of the conventional procedure. Sung-Jong *et al.* (25) in turn evaluated the temperature change during low-speed drilling (50 rpm) using infrared thermography in pig ribs. The drilling technique did not produce heat exceeding 47ºC, which is the critical temperature for bone necrosis during low-speed drilling (7); thus, low-speed drilling without irrigation could be used during implant site preparation.

It seems evident that when a drilling protocol is used without irrigation, the drilling speed must be lowered to reduce friction, minimizing the temperature increase in the bone and thus avoiding thermal injury. In the present study, speeds of 800 rpm were used in the group of implants placed with irrigation, versus 50 rpm in the implants placed without irrigation. Most of the studies on bone overheating are made *in vitro*, mainly using two measurement methods: thermal chambers and thermocouples (26). These measurement systems cannot be applied *in vivo*, though other signs such as implant survival rate or peri-implant bone loss could be useful for analyzing the clinical impact of these drilling techniques in the implant bed of the patient. The principal outcome addressed in the present study were marginal bone loss 12 months after loading. Peri-implant marginal bone level was assessed using parallelized periapical radiographs. Mean bone loss after 12 months of follow-up was 0.83±0.73 mm in the irrigation group and 0.70±0.62 mm in the non-irrigation group. No statistically significant differences were observed between irrigation and non-irrigation implant placement. No *in vivo* studies have evaluated the relationship between peri-implant bone loss and low-speed drilling. In group A, a peri-implant bone loss of 0.83±0.73 mm was observed, coinciding with the observations of a recent systematic review (27) of platform switching with conventional drilling, which demonstrated a range of marginal bone-level changes between 0.055-0.99 mm. On the other hand, there are several studies corresponding to a brand of implants in which low-speed drilling without irrigation is specified in the surgical protocol, with an implant success rate of 97.3% after 5 years of follow-up (28). Al-Hashedi *et al.* (29) compared a type of implant placed with conventional drilling versus another implant placed with low-speed drilling without irrigation. The authors concluded that both implants demonstrated similar peri-implant soft tissue and alveolar bone changes.

In the present study, increased bone loss was observed in those implants placed in the mandible, regardless of the drilling technique used, and although these differences were not statistically significant given the limited sample size, the result suggests a very weak tendency (*p* = 0.165). These findings could be explained by the fact that thermal conductivity varies between cortical and cancellous bone, probably because of the different rate of vascular penetration (15). In a histological assessment of the effect of osteotomy in both types of bone, Stelzle *et al.* (4) recorded the highest temperature in the cortical areas.

Despite de reduced sample size (25 patients and 30 implants), the present study adds to the available evidence regarding the non-irrigation technique. The study comprised 25 consecutive patients selected on the basis of strict, uniform criteria and treated by the same team of professionals using exactly the same procedures. Within its limits, the present study suggests that the low-speed drilling technique without irrigation offers peri-implant bone loss outcomes similar to those of the conventional drilling technique. Further clinical studies with longer follow-up times and larger samples are needed to better understand the influence of the drilling technique (conventional speed with irrigation or low-speed without irrigation) upon peri-implant bone behavior, and to analyze the effects of the temperature rise.
